# Reallocating just 10 min to moderate-to-vigorous physical activity from other components of 24-hour movement behaviors improves cardiovascular health in adults

**DOI:** 10.1186/s12889-024-19255-6

**Published:** 2024-07-03

**Authors:** Yemeng Ji, Muhammed M. Atakan, Xu Yan, Jinlong Wu, Jujiao Kuang, Li Peng

**Affiliations:** 1 Physical Education College, Southwest University, Chongqing, 400715 China; 2 Division of Nutrition and Metabolism in Exercise, Faculty of Sport Sciences, Hacettepe University, Ankara, 06800 Turkey; 3 Institute for Health and Sport, Victoria University, Melbourne, 14428 Australia

**Keywords:** Cardiovascular health, Compositional data analysis, Isotemporal substitution, Moderate-to-vigorous physical activity

## Abstract

**Background:**

As components of a 24-hour day, sedentary behavior (SB), physical activity (PA), and sleep are all independently linked to cardiovascular health (CVH). However, insufficient understanding of components’ mutual exclusion limits the exploration of the associations between all movement behaviors and health outcomes. The aim of this study was to employ compositional data analysis (CoDA) approach to investigate the associations between 24-hour movement behaviors and overall CVH.

**Methods:**

Data from 581 participants, including 230 women, were collected from the 2005–2006 wave of the US National Health and Nutrition Examination Survey (NHANES). This dataset included information on the duration of SB and PA, derived from ActiGraph accelerometers, as well as self-reported sleep duration. The assessment of CVH was conducted in accordance with the criteria outlined in Life’s Simple 7, encompassing the evaluation of both health behaviors and health factors. Compositional linear regression was utilized to examine the cross-sectional associations of 24-hour movement behaviors and each component with CVH score. Furthermore, the study predicted the potential differences in CVH score that would occur by reallocating 10 to 60 min among different movement behaviors.

**Results:**

A significant association was observed between 24-hour movement behaviors and overall CVH (*p* < 0.001) after adjusting for potential confounders. Substituting moderate-to-vigorous physical activity (MVPA) for other components was strongly associated with favorable differences in CVH score (*p* < 0.05), whether in one-for-one reallocations or one-for-remaining reallocations. Allocating time away from MVPA consistently resulted in larger negative differences in CVH score (*p* < 0.05). For instance, replacing 10 min of light physical activity (LPA) with MVPA was related to an increase of 0.21 in CVH score (95% confidence interval (95% CI) 0.11 to 0.31). Conversely, when the same duration of MVPA was replaced with LPA, CVH score decreased by 0.67 (95% CI -0.99 to -0.35). No such significance was discovered for all duration reallocations involving only LPA, SB, and sleep (*p* > 0.05).

**Conclusions:**

MVPA seems to be as a pivotal determinant for enhancing CVH among general adult population, relative to other movement behaviors. Consequently, optimization of MVPA duration is an essential element in promoting overall health and well-being.

## Background

Daily behaviors are inseparably bound to individuals’ cardiovascular health (CVH). As components of movement behaviors, the independent associations of sedentary behavior (SB), physical activity (PA), and sleep with CVH have been well established [1–4]. However, it is essential to recognize that SB, PA, and sleep collectively constitute a fixed 24-hour period, with each component interrelating and interacting with others, suggesting that changes in one component are likely to have a substantial effect on the associations between other components and health outcomes. Therefore, studies in isolation may lead to underestimation of the true associations between movement behaviors and CVH.

In recent years, there has been growing acknowledgement of the feature of perfect multicollinearity among 24-hour movement behaviors and the limitations of the previous paradigm [5, 6]. Following the introduction of the isotemporal substitution model (ISM) by Mekary and colleagues into the field of physical activity epidemiology [7], SB, PA, and sleep have been validated as a finite whole, and this new paradigm has gained widespread application [8–10]. Furthermore, the ISM has undergone continuous refinement [11, 12], and has evolved into a more scientifically rigorous method known as compositional data analysis (CoDA) [13, 14].

Numerous studies have employed CoDA to investigate the associations between movement behaviors and CVH. For instance, Farrahi et al. [15] utilized this approach and confirmed that components of 24-hour movement behaviors were significantly associated with cardiometabolic outcomes, including fasting plasma glucose (FPG) and blood lipid, among middle-aged Finnish adults. Similarly, another study verified a strong association between movement behaviors during the waking day and cardiometabolic biomarkers in children and youth aged 6–17 years [16]. However, despite the abundance of existing studies [15–20], researchers have primarily concentrated on examining the separate factors of cardiovascular risk, particularly the biochemical markers, while neglecting a comprehensive assessment of CVH. It is critical to realize that daily behaviors, such as smoking and unhealthy diets, also have important implications for CVH [21], and should be taken into consideration. “Life’s Simple 7” (LS7), proposed by the American Heart Association (AHA) [22], provides a comprehensive framework to assess overall CVH by considering both health behaviors and health factors, therefore, has become one of the most widely adopted evaluation criteria [23, 24].

Given the comparative lack of findings concerning the associations between 24-hour movement behaviors and overall CVH as determined by CoDA, this study had a two-fold aim: first, to examine the association between 24-hour movement behaviors and CVH score, including the relative associations of individual SB, PA, and sleep with CVH score, evaluated according to LS7’s criteria; second, to explore the estimated differences in CVH score resulting from reallocating fixed time from one component to another, or to the remaining movement behaviors.

## Methods

### Study design and participants

Data were collected from the National Health and Nutrition Examination Survey (NHANES) 2005–2006, a cross-sectional study that used a stratified, multistage probability design to obtain a large, ethnically diverse representative sample of the USA civilian noninstitutionalized population [25]. Detailed study methods can be found at: https://wwwn.cdc.gov/nchs/nhanes/continuousnhanes/default.aspx?BeginYear=2005. Data including demographic data, dietary data, examination data, laboratory data, and questionnaire data were collected through a household interview and a visit to a mobile examination center [26]. Publicly available data were used for this study as a secondary analysis only. In compliance with the Declaration of Helsinki, the protocols of the original study were approved by the Ethics Review Board of the National Center for Health Statistics (#2005-06), and written informed consent was obtained from participants.

### Measurement of 24-hour movement behaviors

Data from the 2005–2006 wave were selected due to the inclusion of accelerometer measurements and a sleep questionnaire. ActiGraph AM-7164 (Pensacola, FL, USA) accelerometers were used to objectively access time spent in SB and PA. Except when sleeping or water-based activities, participants wore the device recorded at 100 Hz on the waist over 7 consecutive days. Data with at least 4 days and ≥ 10 h/d of wear time were considered valid [27]. Using standard count per minutes (cpm) thresholds each 1-min epoch was classified as SB (< 100 cpm), light physical activity (LPA) (100 to 2020 cpm) or moderate-to-vigorous physical activity (MVPA) (> 2020 cpm) [28, 29]. Non-wear time was defined if cpm was 0 for more than 60 consecutive minutes, with allowance for up to 2 min of counts between 1 and 100 [29]. Sleep duration was counted as a whole integer from 1 to 12 (over 12 h were treated as 12), depending on the response to the question “How much sleep do you usually get at night on weekdays or workdays?”. Time spent in each component of 24-hour movement behaviors was tallied daily, averaged across all valid days, and expressed as a proportion of 24 h [11].

### Assessment of CVH metrics and calculation of CVH score

Life’s Simple 7, published by the AHA to systematically assess CVH, consists of health behaviors and health factors [22]. Health behaviors contain smoking status, PA duration, healthy diet and body mass index (BMI). Health factors comprise 3 cardiometabolic risk factors: blood pressure (BP), total cholesterol (TC), and FPG. PA was intentionally omitted from the computation of CVH score as movement behaviors were the primary focus of interest in this analysis [30]. CVH score was evaluated according to the definitions of LS7 [31], and each metric was further categorized as ideal, intermediate, and poor, and given a point score of 2, 1, or 0, respectively. Subsequently, all the points were totaled, with “10 to 12 points” signifying an ideal CVH, “6 to 9 points” indicating an intermediate score, and “0 to 5 points” denoting a poor score [23]. The total CVH score was considered as continuous variable because of its sensitivity and vulnerability to errors [32].

Smoking status was determined based on responses to questions from the cigarette use questionnaire, which included inquiries such as “Do you now smoke cigarettes?” and “How long since quit smoking cigarettes?”. Participants who never smoked obtained a score of 2, 1 for former smokers who had quit more than 12 months, otherwise, 0 point. The dietary score was derived from responses to a 139-question food frequency questionnaire, on basis of which we integrated the average intake of added-sugar and sodium from two 24-hour diet recall. Due to a lack of complete information on sugar-sweetened beverage consumption, added-sugar intake was utilized instead [24]. Participants received 2 points if meeting four or five of the following 5 ideal dietary recommendations, score of 1 if meeting two or three, and 0 points for meeting one or zero [22]: fruits and vegetables ≥ 4.5 cups per day, fiber-rich whole grains ≥ 3 servings per day, fish ≥ 2 servings per week, sodium < 1500 mg/day, added sugar < 37.5 g/day for men, < 25 g/day for women. The weight and height of participants were measured by trained personnel during examination to calculate BMI. BMI < 25 kg/m2 was considered as ideal, 25 ≤ BMI < 30 kg/m2 indicated intermediate status, ≥ 30 kg/m2 categorized as poor [33].

Resting BP was recorded 3 to 4 times and the final values of systolic blood pressure (SBP) and diastolic blood pressure (DBP) were computed from the average of 2 readings, omitting the questionable values. Self-reported use of antihypertensive medications and questionnaire of prescription medications were collected to assist in determining BP categories. Without treatment, BP readings of < 120/80 mmHg were regarded as ideal and corresponded to a score of 2, participants whose SBP was between 120 and 139 mmHg and/or DBP was between 80 and 89 mmHg, or those whose BP was effectively managed with treatment, were classified as intermediate and awarded 1 point. BP readings ≥ 140/90 mmHg were categorized as poor and resulted in a score of 0. Laboratory values of TC and FPG were collected from blood samples in the mobile examination center, and medication use for both were obtained from the same questionnaire of prescription medications. Untreated TC < 200 mg/dl was categorized as ideal, TC in the range of 200 to 239 mg/dl or treatment to target was regarded as intermediate, ≥ 240 mg/dl indicated poor. FPG was categorized as ideal (< 100 mg/dl without treatment), intermediate (100 − 125 mg/dl or treated to goal), or poor (> 125 mg/dl).

### Covariates

To control for confounding effects, the following variables, including age, gender, race, education, marital status, household income, employment status, and alcohol consumption were regarded as covariables based on the classification of original data in NHANES 2005–2006. The socio-demographic data were obtained through household interview, employment status was determined by answers to “Type of work done last week” and “Main reason did not work last week”, and the frequency of alcohol consumption was evaluated by alcohol use questionnaire. The socio-demographic variables were selected based on previous research on their strong relationships with CVH metrics [34–36], besides, employment status and alcohol consumption were controlled as covariates due to the significant associations with CVH score [37, 38]. All confounders were treated as categorical variables with exception of age as continuous variable in the model.

### Statistical analysis

IBM SPSS Statistics Version 26 (IBM Corp., Armonk, NY, USA) served to produce descriptive statistics. Continuous variables were characterized by mean ± standard deviation, while categorical variables were represented as percentage. CoDA were performed using the “compositions” [39], “robCompositions” [40], and “lmtest” packages in Rstudio 2022.12.0 (The R Foundation for Statistical Computing, Vienna, Austria). The central tendency of 24-hour movement behaviors was expressed as compositional mean, with the durations of SB, LPA, MVPA, and sleep being adjusted linearly to ensure that the total sum of time-use compositions equaled 1440 min. The dispersion was described by the compositional variation matrix, in which the higher value indicates the lower probability of co-dependence between two components.

The principle of CoDA is to explain the associations of components with outcome variables by transforming the values of components from arithmetic mean to geometric mean, thus transforming the data from restricted simplex space to standard real space, then analyzing by multiple linear regression analysis, and finally inverting to form the regression coefficients in simplex space. The sequential binary partition was introduced into the above analysis process to randomly allocate two components of 24-hour movement behaviors to the numerator of the first isometric log-ratio (ilr) coordinate, while the rest two components were assigned to the denominator. The method for constructing the complete set of ilrs coordinates was described in detail elsewhere [41]. As explanatory variables, the ilrs coordinates and covariables were taken to fit the compositional multiple linear regression model, which intended to display the association between all time-use compositions and CVH score. The model’s linearity, including quadratic terms for the ilrs, and normality were checked [42, 43], and the significance was examined using the “car::Anova” function. Subsequently, in accordance with the procedure in study by Dumuid et al. [13], each component of 24-hour movement behaviors was consecutively transformed into the numerator of the first ilr coordinate, thereby representing its relative association in relation to the health outcome. Consequently, four ilr coordinate systems were established. The relative associations of each component compared to other three with CVH score were ascertained by fitting 4 separate multiple linear regression models. The basic parameters for the first ilr coordinate of 4 models were reported, including the beta coefficients, standard error (SE) of beta, t-statistic, and p-values.

On basis of the above analyses, the principle of isotemporal substitution was adopted to predict the differences in CVH score when reallocating fixed time (in 10-mintue increments within 60 min) between SB, LPA, MVPA, and sleep while compositional mean of 24-hour movement behaviors was set as the initial value. 10 min was chosen as the unit of reallocation according to its health effect [11, 44]. The reallocations away from remaining movement behaviors proportionally to one component were conducted firstly (referred to as one-for-remaining reallocations). The duration of one component in the numerator of the first ilr coordinate was changed, and time spent in remaining movement behaviors in the denominator was corresponding altered in proportion to maintain a total of 1440 min, and to predict the differences in CVH scores. Furthermore, equivalent procedures were executed for one-for-one reallocations, where one component of 24-hour movement behaviors was substituted for another, while maintaining the constancy of the other two behaviors. The statistical significance was established at *p* < 0.05.

## Results

### Descriptive statistics

**Fig. 1:**
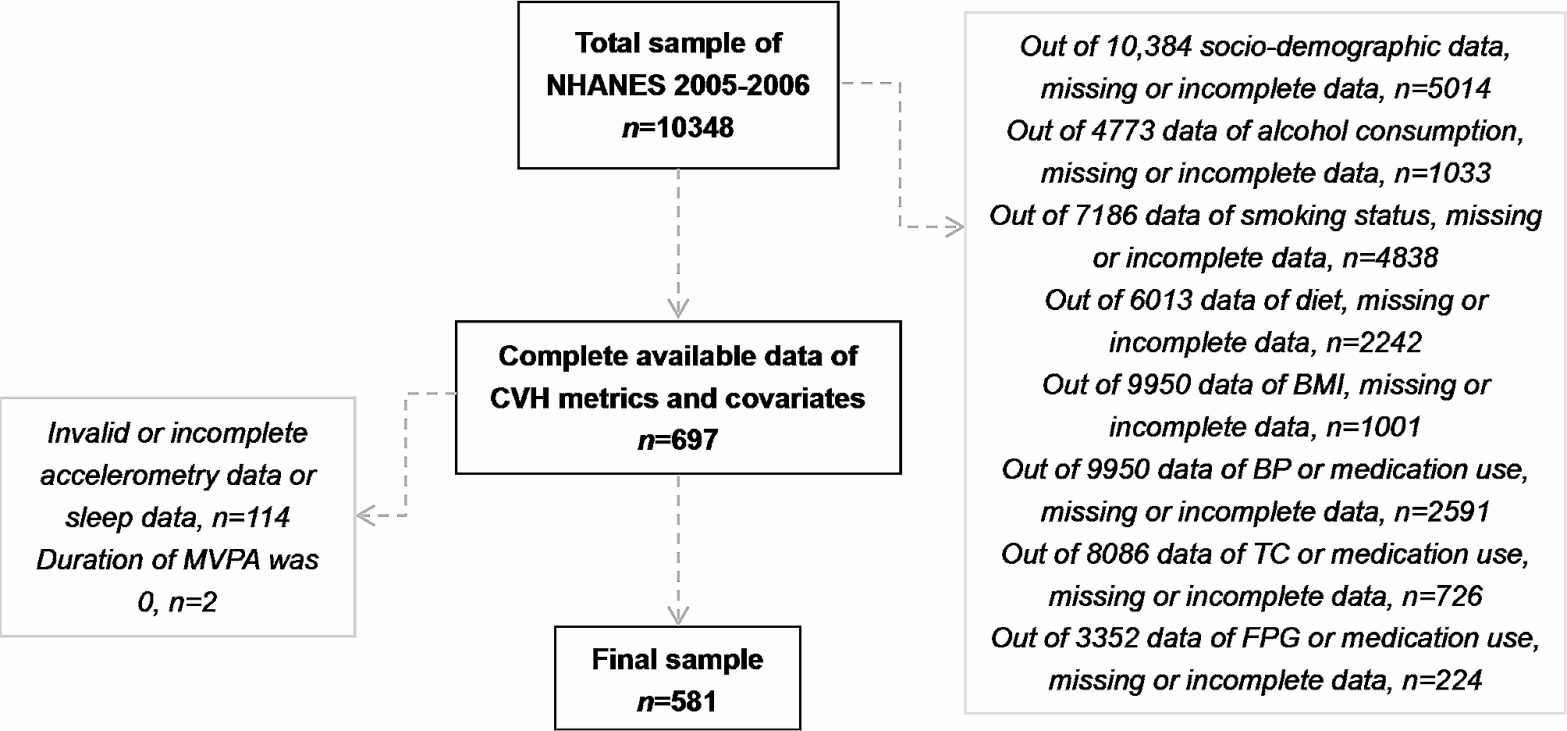
Participant flow. *BMI* body mass index, *BP* blood pressure, *CVH* cardiovascular health, *FPG* fasting plasma glucose, *MVPA* moderate-to-vigorous physical activity, *NHANES* National Health and Nutrition Examination Survey, *TC* total cholesterol

Participants who were younger than 20 years, pregnant or breastfeeding at the time of survey, or missing any of the required data were excluded. Eventually, a total of 581 participants from the NHANES 2005–2006 dataset met the inclusion criteria for this study and were consequently included in the analysis. Figure 1 shows the participant flow. Table 1 presents the demographic characteristics of the included participants, and Table 2 illustrates the distribution characteristics of CVH score.

**Table 1:** Descriptive characteristics of demography for the final sample from NHANES (*n* = 581)

Covariates	Category	Number (%) or Mean ± SD
**Age**		54.9 ± 16.8
**Gender**, ***n*(%)**	*Male*	351 (60.4%)
	*Female*	230 (39.6%)
**Race**, ***n*(%)**	*Mexican American*	89 (15.3%)
	*Other Hispanic*	12 (2.1%)
	*Non-Hispanic White*	352 (60.6%)
	*Non-Hispanic Black*	103 (17.7%)
	*Other Race, Including Multi-Racial*	25 (4.3%)
**Education**, ***n*(%)**	*Less Than 9th Grade*	51 (8.8%)
	*9-11th Grade (Includes 12th grade with no diploma)*	98 (16.9%)
	*High School Graduate*	165 (28.4%)
	*Some College or AA degree*	166 (28.6%)
	*College Graduate or above*	101 (17.4%)
**Marital status**, ***n*(%)**	*Married*	332 (57.1%)
	*Widowed*	52 (9%)
	*Divorced*	69 (11.9%)
	*Separated*	18 (3.1%)
	*Never Married*	57 (9.8%)
	*Living with Partner*	53 (9.1%)
**Household income**, ***n*(%)**	*0-4999$*	17 (2.9%)
	*5000–9999$*	31 (5.3%)
	*10,000–14,999$*	48 (8.3%)
	*15,000–19,999$*	43 (7.4%)
	*20,000–24,999$*	54 (9.3%)
	*25,000–34,999$*	83 (14.3%)
	*35,000–44,999$*	72 (12.4%)
	*45,000–54,999$*	56 (9.6%)
	*55,000–64,999$*	36 (6.2%)
	*65,000–74,999$*	33 (5.7%)
	*Over 75,000$*	108 (18.6%)
**Employment status**, ***n*(%)**	*Work now*	314 (54%)
	*Not Work now*	267 (46%)
**Alcohol consumption**, ***n*(%)**	*< 1 time/month*	380 (65.4%)
	*≥ 1 time/month*	201 (34.6%)

**Table 2:** Descriptive characteristics of CVH score for the study population (*n* = 581)

CVH metrics	Category	Number (%) or Mean ± SD
**Smoking status**, ***n*(%)**	*Poor*	226 (38.9%)
	*Intermediate*	27 (4.6%)
	*Ideal*	328 (56.5%)
**Healthy diet**, ***n*(%)**	*Poor*	477 (82.1%)
	*Intermediate*	104 (17.9%)
	*Ideal*	0 (0.0%)
**BMI, kg/m** ^**2**^ **BMI**, ***n*(%)**		28.61 ± 6.37
	*Poor*	204 (35.1%)
	*Intermediate*	195 (33.6%)
	*Ideal*	182 (31.3%)
**BP**, ***n*(%)**	*Poor*	136 (23.4%)
	*Intermediate*	338 (58.2%)
	*Ideal*	107 (18.4)
**SBP, mmHg**		126.67 ± 18.53
**DBP, mmHg**		69.31 ± 12.05
**TC, mmol/L** **TC**, ***n*(%)**		200.07 ± 44.76
	*Poor*	100 (17.2%)
	*Intermediate*	267 (46%)
	*Ideal*	214 (36.8%)
**FPG, mmol/L** **FPG**, ***n*(%)**		106.63 ± 31.01
	*Poor*	68 (11.7%)
	*Intermediate*	220 (37.9%)
	*Ideal*	293 (50.4%)
**CVH score** **CVH score**, ***n*(%)**		5.85 ± 1.91
	*Poor*	230 (39.6%)
	*Intermediate*	337 (58%)
	*Ideal*	14 (2.4%)

*BMI* body mass index, *BP* blood pressure, *CVH* cardiovascular health, *DBP* diastolic blood pressure, *FPG* fasting plasma glucose, *SBP* systolic blood pressure, *SD* standard deviation, *TC* total cholesterol

Removing non-wear invalid time, the average accelerometer wear time was 1312.2 ± 99.7 min/d. Compositional mean of 24-hour movement behaviors was calculated after linearly adjusting to 1440 min/d. Participants spent an average of 604.1 min/d (42.0%) in SB, 465.6 min/d (32.3%) in sleep, 358.8 min/d (24.9%) in LPA, and 11.5 min/d (0.8%) in MVPA, respectively. While around 33.2% of the included participants met the recommended MVPA duration (≥ 21.4 min/d [45]), more than 71.3% of individuals spent over 8 h per day in SB. Table 3 shows the variability of compositional data, the values represent the log variance of two components of 24-hour movement behaviors. The smallest variance of log (sleep/LPA) indicated that time spent in sleep was highly co-dependent with LPA. The highest log-ratio variances were observed for MVPA, demonstrating the least likelihood of conversion for MVPA with other components.

**Table 3:** Compositional variation matrix of 24-hour movement behaviors

	LPA	MVPA	SB	Sleep
**LPA**	0.000000	1.581860	0.16325	0.083061
**MVPA**	1.581860	0.000000	2.06873	1.805471
**SB**	0.163255	2.068731	0.00000	0.109166
**Sleep**	0.083061	1.805471	0.10917	0.000000

The values represent the log-ratio variance of each two components. *LPA* light physical activity, *MVPA* moderate-to-vigorous physical activity, *SB* sedentary behavior

### Compositional regression analyses

The results from compositional multiple linear regression showed that the first ilr coordinate of 24-hour movement behaviors was strongly associated with CVH score (*p* < 0.00001), and among all covariables, gender, education (*Some College or AA degree* and *College Graduate or above*), and marital status (*Divorced*) were significantly associated with CVH score (*p* < 0.05). Although the effect size (ES) was relatively small (R²adj = 0.1036), 24-hour movement behaviors emerged as a significant predictor of CVH score. No quadratic relationships between time-use compositions and CVH score existed (*p* > 0.05). Since the regression coefficient for the first ilrs in the model could not directly reflect the relative associations of individual components with health outcome [13], 4 models were then conducted to obtain 4 regression coefficients for the first ilr of each component relative to others. The beta coefficients and p-values for 4 models are reported in Table 4. The results showed that the first ilr coefficient of Model 2 was positive and *p* < 0.001, indicating a significant positive association between MVPA (relative to remaining movement behaviors) and CVH score. By contrast, the p-values for Model 1, Model 3, and Model 4 emerged as greater than 0.05, suggesting that SB, LPA, and sleep lacked significant relative associations with CVH score.

**Table 4:** Results of four multiple linear regression models for the first ilr

Model	Each component relative to remaining	Beta coefficients for ilr1	SE	t-value	*p*-value
**Model 1**	LPA: remaining	-0.38	0.24	-1.60	0.11
**Model 2**	MVPA: remaining	0.38	0.09	4.40	< 0.001**
**Model 3**	SB: remaining	-0.36	0.24	-1.52	0.13
**Model 4**	Sleep: remaining	0.36	0.29	1.22	0.22

**p < 0.01. *ilr* isometric log-ratio, *LPA* light physical activity, *MVPA* moderate-to-vigorous physical activity, *SB* sedentary behavior, *SE* standard error

### Compositional isotemporal substitution analyses: one-for-remaining reallocations

**Fig. 2:**
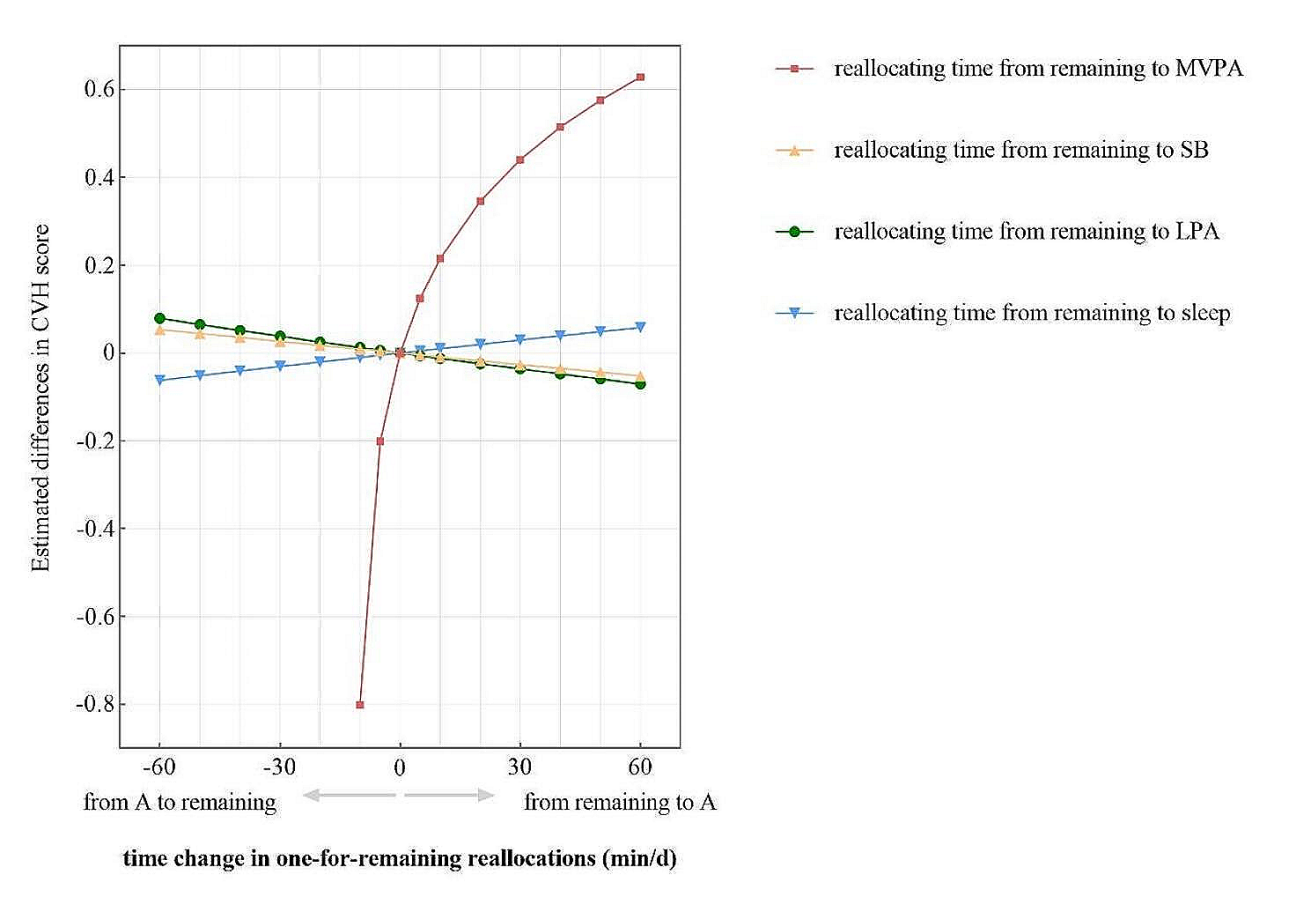
Estimated differences in CVH score associated with time reallocations from remaining components of 24-hour movement behaviors in proportion to one component. *CVH* cardiovascular health, *LPA* light physical activity, *MVPA* moderate-to-vigorous physical activity, *SB* sedentary behavior

Figure 2 presents the predicted differences in CVH score when 10 to 60 min were reallocated from individual component to remaining movement behaviors in proportion. Given that compositional data must be non-negative [13] and compositional mean of MVPA was 11.5 min/d, MVPA duration was only increased, but not decreased when reallocations of time exceeded 10 min. As the dominant component of reallocations, an increase in MVPA (accompanied by the proportionate decrease in other components) exhibited a significant association with a positive change in CVH score, displaying a curve increasing trend. Meanwhile, the variations in CVH score were not equivalent when MVPA replaced other movement behaviors in proportion or was replaced with the same reallocation time, indicating an asymmetrical relationship. For example, reallocating 10 min away from remaining movement behaviors to MVPA, there was an associated increase of + 0.22 in CVH score (95% confidence interval (95% CI) 0.12 to 0.31). Conversely, reallocating the same 10 min duration from MVPA resulted in a decrease of 0.80 in CVH score (95% CI -1.16 to -0.44). There were no statistically significant differences in CVH score when LPA, SB, and sleep were the dominant component in reallocations (*p* > 0.05).

### Compositional isotemporal substitution analyses: one-for-one reallocations

**Fig. 3:**
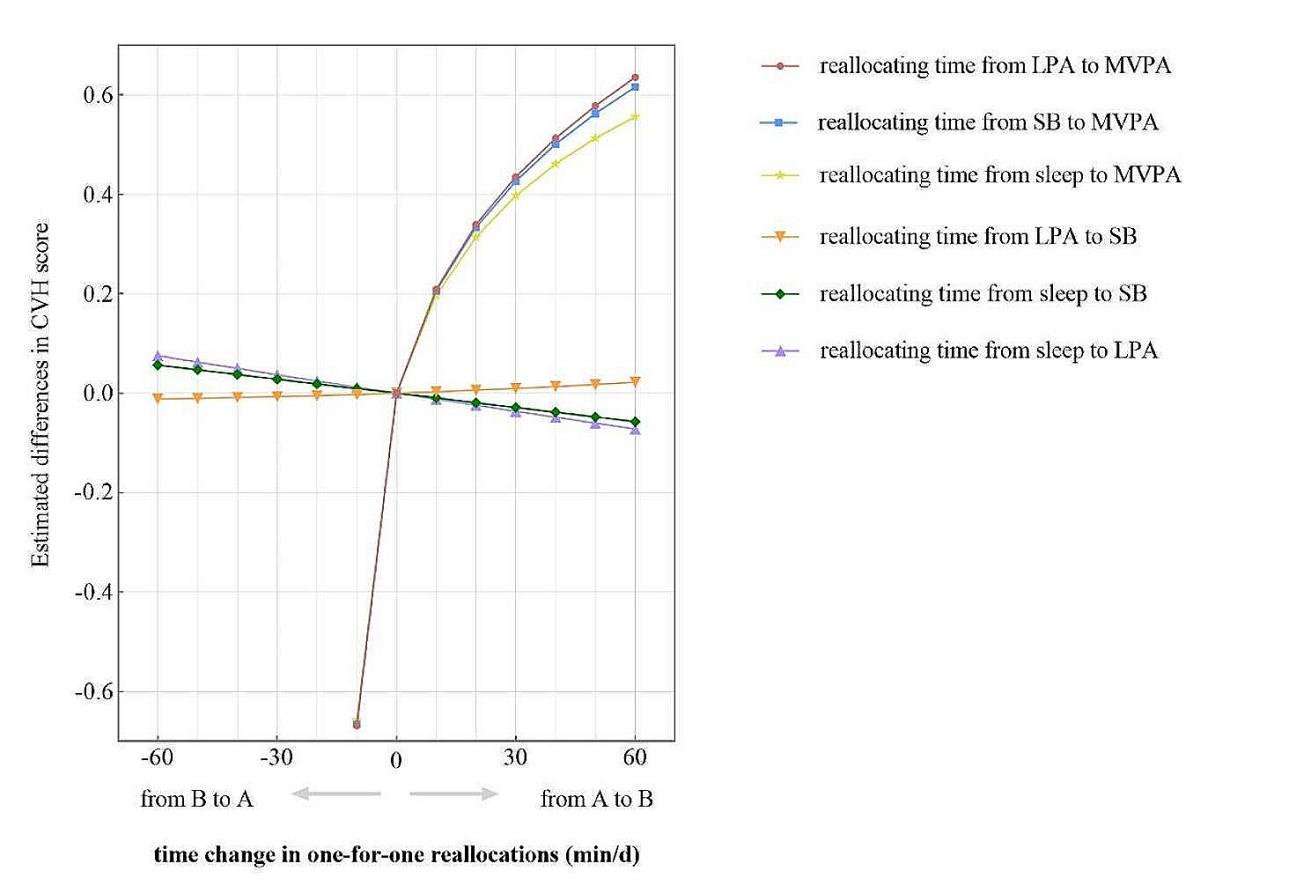
Estimated differences in CVH score associated with time reallocations from one component to another, keeping the rest two constant. *CVH* cardiovascular health, *LPA* light physical activity, *MVPA* moderate-to-vigorous physical activity, *SB* sedentary behavior

Keeping the rest two components at the compositional mean, we systematically reallocated time (in the unit of 10 min) from one component of 24-hour movement behavior to another in turn, in order to predict the differences in CVH score. The results demonstrated that all compositional isotemporal substitutions involving MVPA were significantly associated with overall CVH (*p* < 0.05), similarly to the findings for one-for-remaining reallocations. Generally, there were favorable improvements in CVH score when MVPA replaced other three components, but the rate of increase in CVH score gradually diminished with continuous maximization of MVPA duration. The differences in CVH score were slightly predominant when MVPA replaced LPA (ES = 0.64 for 60 min, *p* < 0.05), compared to the same time reallocations from either SB or sleep (ES = 0.62/0.56 when MVPA replaced SB/sleep, *p* < 0.05). In addition, the asymmetry of predicted differences in CVH score persisted in one-for-one reallocations when MVPA replaced other components or was replaced, but the magnitudes of negative change were generally smaller than values in one-for-remaining reallocations. For example, when 10 min was reallocated from MVPA to LPA, SB, or sleep, a decrease of 0.67/0.67/0.66 points was observed in CVH score (95% CI -0.99 to -0.35/ -0.98 to -0.35/ -0.97 to -0.34), smaller than − 0.80 when MVPA was replaced with remaining components (95% CI -1.16 to -0.44). However, reallocations of the same time from other individual components to MVPA subsequently resulted in increase of 0.21/0.21/0.20 (95% CI 0.11 to 0.31/ 0.11 to 0.30/ 0.10 to 0.29), resembling the effects in one-for-remaining reallocations (95% CI 0.12 to 0.31). Again, no statistical significance was observed in CVH score when reallocations only involved LPA, SB, and sleep (*p* > 0.05). All results for one-for-one reallocations are depicted in Fig. 3.

## Discussion

The primary finding of this cross-sectional study indicated a significant association between 24-hour movement behaviors as a whole and overall CVH in general adult population. Moreover, reallocations of fixed time to MVPA from other components individually or proportionally from remaining movement behaviors were consistently associated with beneficial CVH. Our study is novel for setting CVH score, calculated based on the criteria in LS7, as the health outcome. Additionally, we employed CoDA approach to investigate the combined and relative associations of 24-hour movement behaviors and each component with overall CVH. Furthermore, compositional isotemporal substitution was adopted to predict the differences in overall CVH resulting from theoretical reallocations.

The results of this study aligned with previous research, which emphasized the importance of thoroughly evaluating cardiometabolic health and utilized a calculated score of individual cardiometabolic risk factors to explore the relationships between movement behaviors and heart health. For instance, Simone et al. [46] employed CoDA and discovered a notable association between time-use compositions (including 9 components: time in shorter and longer bouts of sedentary behavior; time in shorter and longer bouts of light-, moderate-, or vigorous-intensity PA; other time) and cardiometabolic risk score (CMR score) among 782 youths aged 7–13 years, where CMR score was assessed based on data of BP, waist circumference, lipoprotein cholesterol, and triglycerides. Several studies focused on metabolic syndrome score, which was computed based on similar risk factors, and demonstrated the correlations between movement behaviors and score in various age groups including children, adults, and the elderly [47, 48]. Remarkably, most of these studies neglected the potential implications of daily behaviors such as diet and smoking, even only few studies controlled them as covariates. In light of the limitations of such research, we combined daily behaviors with cardiovascular risk factors, resulting in the derivation of a comprehensive CVH score, and revealed the association between 24-hour movement behaviors and overall CVH in adults.

The findings of our study on the differences in health outcomes caused by the reallocations of fixed time between movement behaviors were partially in line with the findings of German et al. [30], who firstly used CVH score as a measure of health outcome and performed isotemporal substitution analyses using the non-CoDA approach. The results related to MVPA exhibited consistency across both studies. German et al. [30] found that substituting 30 min of SB with MVPA was associated with a 0.485-point increase in CVH score, close to the 0.427 increase in our study (95% CI 0.23 to 0.62). However, the results pertaining to other components were mixed. German and colleagues observed a favorable association of a 0.077 rise in CVH score when replacing 30 min of SB with sleep, whereas our study found a modest and statistically insignificant increase in CVH score (ES = 0.028, 95% CI -0.02 to 0.08) when 30 min SB were replaced with sleep. In terms of reallocations between LPA and SB, radically distinct results were observed in two studies. Specifically, the investigators observed a significant increase in CVH score when 30 min LPA substituted for SB (ES = 0.039) [30], but our study suggested that the association was opposite and insignificant (ES=-0.007, 95% CI -0.05 to 0.03). The discrepancy between the results of two studies may be attributed to various reasons, including the followings: First, the types of ISM. The accuracy of obtained results may be compromised [5] because of the failure to treat movement behaviors as compositional data during the analysis using traditional ISM [7]. Second, the diversity of participants should not be ignored. In comparison to the broader adult population (aged 20–85 years) included in our study, participants in their study were middle-age and older adults (aged 45–84 years), with a higher average age. It is worth noting that for the elderly, engaging in LPA yields comparatively greater advantages when reallocating equivalent time from SB [19, 49], which may result in disparities in findings. Third, the initial baseline levels of components have undeniably impact on the correlations. For instance, the average sleep duration of participants before reallocations in our study was longer compared to the study by German et al. Since participants already obtained ample sleep, the favorable influence of increasing sleep duration became less significant when sleep substituted SB. As a result, the predicted positive association was attenuated or even disappeared. Besides, compositional mean of MVPA was lower in our study, thereby leading to stronger associations between isotemporal substitution involving MVPA and CVH score. These findings may reflect the nature of compositional data: A small value in one component leads to a greater variability in response to relative changes [13].

This study provided further evidence supporting the positive association of MVPA with CVH, consistent with the previous literatures [15, 50–53]. In addition, employing CoDA, we found a notable asymmetry of the predicted differences in CVH score when substitution involved MVPA, regardless of in one-for-one reallocations or one-for-remaining reallocations [12, 41, 54, 55]. This characteristic led to the formation of a dose-response curve for MVPA duration [56], illustrating that a sustained increase in MVPA duration cannot generate the benefit with same growth trend [57]. Hence, it is advisable for general population to prioritize maximizing MVPA while also maintaining a high level of MVPA duration. Nonetheless, because the curve did not exhibit a slowing or decreasing trend, it is possible that we were unable to determine the exact duration of MVPA that would provide the greatest CVH benefits [43], so further research is required. The results for the relative associations between substitution with LPA and CVH were controversial in prior research [46, 48, 56, 58], which was in line with our finding that did not appreciably demonstrate the significant associations with CVH score when LPA replaced other components or was replaced. This finding implies that the intensity of PA may have a more significant impact on overall CVH compared to its duration.

The association of sleep as a component of 24-hour movement behaviors with CVH has garnered growing interest in recent years. A few studies identified the U-shaped association between sleep and various health outcomes [15, 17, 42, 59]. Nevertheless, considering that most participants in our study reported sleep duration within the range of 7–9 h, with only 1.9% exceeding 9 h per day, it was reasonable to conclude that such a relationship did not exist in this study. More importantly, self-reported sleep duration was prone to recall biases, which may affect the accuracy of relevant results. Further efforts should be made to measure sleep in more precise ways and to explore the association of sleep as a component with CVH in populations with sleep problems, such as sleep-deprived workers or people with circadian reversal.

### Strengths and limitations

A key strength of this study lies in its utilization of CoDA to further investigate the association between 24-hour movement behaviors and overall CVH in general adult population.

The limitations of this study need to be considered. First, causal inferences between time-use compositions and CVH score cannot be established due to the cross-sectional nature of NHANES 2005–2006. Second, while main confounders such as socio-demographic variables were adjusted for during analyzing, possible residual confounding by other unmeasured factors could not be ruled out. Third, participants lacking any information on CVH metrics were excluded, resulting in a reduced sample size of only 581 participants meeting the inclusion criteria. Consequently, there may be some degree of bias in the results. Fourth, participants in our study had a wide age-span (from 20 to 85 years) and thus the results, such as compositional mean of 24-hour movement behaviors, merely reflected a relative indication of the general adult population, cannot be directly interpreted as an absolute representation of the specific population. Fifth, sleep duration was not precise to the minute due to self-reporting; therefore, cognitive bias may have led individuals to report longer sleep durations than the actual time slept [60]. However, in this study, we observed that some participants continued to wear the accelerometer while sleeping. Therefore, we adjusted the sleep duration for this group by incorporating triaxial data during data categorization, thereby mitigating participants’ bias in reporting sleep duration to some extent. Sixth, dietary intake data for grains, vegetables, fruits, fish, and other food items were collected via the NHANES food questionnaire. This questionnaire assesses the frequency of intake of each food item over a specified period, rather than querying participants about the specific weight of the food items they consume daily. Consequently, in this study, dietary scores were computed based on the daily frequency of intake of various food types, deviating from the calculation method utilized in prior studies. Moreover, it is noteworthy that the dietary score may potentially be inaccurate, as the questionnaire might not encompass all food types. Lastly, the limitations of waist-worn accelerometers in accurately distinguishing standing or sitting postures, coupled with the definition of SB set at 100 cpm, may lead to an underestimation of the actual duration spent in SB [61]. In addition, the cut points used to define movement behaviors were established based on the characteristics of the general adult population. This may have resulted in an underestimation of MVPA duration among the elderly [62].

## Conclusions

Our study offers compelling evidence of the cross-sectional association between 24-hour movement behaviors and overall CVH in adults. The findings suggested that reallocations of time from any other time-use compositions to MVPA were associated with more favorable CVH, highlighting the importance of MVPA and supporting the inclusion of MVPA in the assessment of CVH in LS7. Meanwhile, the curve positive association between MVPA and CVH score, relative to other components, also suggested that we should maintain the current adequate MVPA duration while optimizing it. These findings demonstrate the potential benefits of optimizing an individual’s daily behaviors to enhance CVH and offer a theoretical basis for public health authorities to implement appropriate measures, such as calling on the public to reallocate sitting time to MVPA, to improve the cardiometabolic health, and promoting the monitoring and regulation of personal behaviors among a wider range of age groups through the publication of a 24-hour activity guideline. Future studies should endeavor to conduct more comprehensive longitudinal investigations within the general population, as well as across various age groups, genders, and special populations, including individuals with obesity. These studies should aim to verify the accuracy of the currently observed cross-sectional associations between 24-hour movement behaviors and CVH through actual follow-up assessments. Furthermore, future research could aim to provide more targeted and specific time management approaches for different populations by establishing optimal time-use zones of CVH scores to target specific metrics of CVH.

## Data Availability

The data of original study are available in the NHANES 2005-2006 repository, [https://wwwn.cdc.gov/nchs/nhanes/continuousnhanes/default.aspx?BeginYear=2005], and the datasets used and/or analyzed during the current study are available from the corresponding author on reasonable request.

## Abbreviations

AHA: American heart association
BMI: Body mass index
BP: Blood pressure
CI: Confidence interval
CoDA: Compositional data analysis
cpm: Count per minutes
CVH: Cardiovascular health
DBP: Diastolic blood pressure
FPG: Fasting plasma glucose
ilr: Isometric log-ratio
LPA: Light physical activity
ISM: Isotemporal substitution model
LS7: Life’s Simple 7
MVPA: Moderate-to-vigorous physical activity
NHANES: National health and nutrition examination survey
PA: Physical activity
SB: Sedentary behavior
SBP: Systolic blood pressure
SE: Standard error
TC: Total cholesterol
